# Higher Levels of Stress-Related Hair Steroid Hormones Are Associated with the Increased SCORE2 Risk Prediction Algorithm in Apparently Healthy Women

**DOI:** 10.3390/jcdd9030070

**Published:** 2022-02-27

**Authors:** Eglė Mazgelytė, Neringa Burokienė, Agata Vysocka, Martynas Narkevičius, Tomas Petrėnas, Andrius Kaminskas, Jurgita Songailienė, Algirdas Utkus, Dovilė Karčiauskaitė

**Affiliations:** 1 Department of Physiology, Biochemistry, Microbiology and Laboratory Medicine, Institute of Biomedical Sciences, Faculty of Medicine, Vilnius University, M. K. Čiurlionio st. 21, LT-03101 Vilnius, Lithuania; dovile.karciauskaite@mf.vu.lt; 2 Clinics of Internal Diseases, Family Medicine and Oncology, Institute of Clinical Medicine, Faculty of Medicine, Vilnius University, M. K. Čiurlionio st. 21, LT-03101 Vilnius, Lithuania; neringa.burokiene@mf.vu.lt (N.B.); agata.janovska@mf.vu.lt (A.V.); martynas.narkevicius@santa.lt (M.N.); 3 Department of Human and Medical Genetics, Institute of Biomedical Sciences, Faculty of Medicine, Vilnius University, M. K. Čiurlionio st. 21, LT-03101 Vilnius, Lithuania; tomas.petrenas@mf.vu.lt (T.P.); andrius.kaminskas@mf.vu.lt (A.K.); jurgita.songailiene@mf.vu.lt (J.S.); algirdas.utkus@mf.vu.lt (A.U.)

**Keywords:** cardiovascular disease risk, chronic stress, hair steroid hormones

## Abstract

Cardiovascular diseases (CVDs) are the major cause of death worldwide. Although the importance of conventional CVD risk factors, including older age, male gender, hypertension, obesity, dyslipidemia and hyperglycemia, is well-studied, psychosocial stress, which is considered an independent CVD risk factor, requires further investigation. Thus, we aimed to investigate the association between long-term secretion of stress-related steroid hormones, including cortisol, cortisone and dehydroepiandrosterone, and the 10-year fatal and non-fatal CVD risk estimated by the SCORE2 risk prediction algorithm, as well as traditional CVD risk factors in a group of apparently healthy women. A total of 145 women (aged 50–64 years) participating in the national CVD prevention program were enrolled in the study. Sociodemographic, lifestyle, health-related characteristics, stress, anxiety and sleep quality indicators were evaluated using specific questionnaires. Anthropometric and arterial blood pressure measures were assessed by trained personnel, lipid and glucose metabolism biomarkers were measured using routine methods, and hair steroid hormone levels were determined by ultra-high-performance liquid chromatography-tandem mass spectrometry. The results showed that higher levels of hair cortisol and cortisone are associated with increased SCORE2 values. Moreover, significant associations between hair glucocorticoids and individual cardiovascular risk factors, including obesity, hypertension, dyslipidemia and hyperglycemia, were found. These findings indicate that stress-related hair steroid hormones might be valuable biomarkers for CVD prediction and prevention.

## 1. Introduction

Cardiovascular disease (CVD) incidence and age-standardized mortality rates are declining in many high-income regions, whereas these only slightly decrease, or at times even increase, in the majority of low- and middle-income countries [1,2]. Globally, CVDs still account for the majority of noncommunicable disease deaths with 17.9 million people affected annually [3]. It is generally accepted that lifestyle risk factors, including unhealthy diet, low physical activity and smoking, are linked to conventional cardiovascular risk factors, such as hypertension, obesity, dyslipidemia and hyperglycemia. Other well-known non-modifiable risk factors are older age, male gender, genetic susceptibility and family history of CVD [1]. Currently, psychosocial stress is considered an independent CVD risk factor: epidemiological data suggest that chronic stress at work and in private life is associated with a 40–50% increased occurrence of coronary heart disease (CHD) [4]. The INTERHEART case-control study, which examined the relationship between modifiable risk factors and the incidence of CHD in 24,767 patients from 52 countries, revealed that self-reported chronic psychosocial stress is associated with a 2.17 times increased myocardial infarction risk after adjusting for traditional cardiovascular risk factors, lifestyle indicators and socioeconomic status [5]. Exposure to chronic stress was related to an increased CVD risk in both men and women, however, it is suggested that women have a higher prevalence of psychosocial factors and are therefore more susceptible and vulnerable to the deleterious effects of stress on CVD [6,7]. Moreover, previous research highlighted the complexities of coronary disease in women, as women tend to have smaller epicardial coronary vessels with more diffuse atherosclerotic disease and are therefore more likely than men to suffer from microvascular coronary disease and endothelial dysfunction [8]. Although women’s enrollment in major cardiovascular randomized clinical trials has increased from 21% between 1986 and 1990 to 33% between 2011 and 2015, it remains lower than the relative proportion in the disease population, and there is a need for specific studies in women [9].

Despite observational evidence of the relationship between chronic psychosocial stress and adverse health effects, the biological mechanism linking stress and somatic diseases has only recently been fully clarified [10]. In brief, the central nucleus of the amygdala is the primary brain structure involved in emotional perception, generation of fear or anger and physiological response to stressors, as its efferent neurons provide input to the stress system, including the hypothalamic–pituitary–adrenal (HPA) axis and locus caeruleus (LC)/norepinephrine (NE) system. Activation of the HPA axis triggers synthesis of corticotropin-releasing hormone (CRH) and arginine vasopressin in the paraventricular nucleus (PVN) of the hypothalamus. CRH binds to the G-protein-coupled receptors in the anterior lobe of the pituitary gland and induces secretion of adrenocorticotropic hormone (ACTH), which stimulates cortisol synthesis in the adrenocortical cells of the zona fasciculata, regulates androgen secretion by the zona reticularis and participates in control of aldosterone secretion by the zona glomerulosa. The upregulation of the LC/NE system leads to the “fight or flight” reaction, due to enhanced synthesis of epinephrine and norepinephrine in the adrenal medulla. The biological effects of these hormones through genomic and non-genomic pathways include increased insulin resistance, blood pressure, heart rate, visceral adiposity, and impaired immune system response, as well as enhanced systemic and arterial inflammation [10,11].

The major obstacle in chronic stress research is the lack of a universal and validated stress measurement tool or biomarker, as distinct studies use different chronic stress evaluation techniques, including: self-reported questionnaires (e.g., Perceived Stress Scale or the self-reported Life Events Checklist) [12,13]; interviews (e.g., UCLA Life Stress Interview) [14]; and allostatic load index calculation [15] or measurement of cortisol in a variety of biological samples [16,17,18]. In the last decade, analysis of cortisol in scalp hair was found to be the most promising methodological approach for assessing chronic stress level. The main advantages of using hair cortisol as a biomarker of chronic stress compared with other specimens (i.e., blood serum, saliva, urine) are: the fairly predictable growth rate (1 cm/month) that allows retrospective assessment of integrated cortisol secretion; the non-invasive sample collection procedure and small amount of sample required for analysis; the easy sample storage, as samples can be stored at room temperature over long periods of time; and the lack of situational confounding factors, such as circadian rhythmicity, acute stress, smoking, alcohol consumption, food intake and intensive physical exercise [17,19]. Previous studies showed that an increased hair cortisol concentration is associated with long-term unemployment [20], shift work [21], severe chronic pain [22], sleep disorders [23] and psychopathologies, including post-traumatic stress disorder [24], major depression [25] and bipolar disorder [26]. Other, less extensively studied steroid hormones—cortisone and dehydroepiandrosterone (DHEA)— might also be considered stress biomarkers. As mentioned earlier, stress-triggered activation of the HPA axis induces a release of cortisol, which is converted to its inactive form, cortisone, by the enzyme 11-beta-hydroxysteroid dehydrogenase type 2 (11β-HSD2), though cortisone can be converted back to cortisol by the 11-beta-hydroxysteroid dehydrogenase type 1 (11β-HSD1). Thus, simultaneous measurement of hair cortisol and cortisone levels can provide comprehensive information about the cumulative amounts of both active and inactive glucocorticoids in the body [27]. Findings on the use of DHEA as a psychosocial stress biomarker are conflicting, as it was shown that under acute stress conditions ACTH induces DHEA secretion from the zona reticularis, while, at the same time, DHEA plays a protective role acting as an antagonist of glucocorticoids [28]. Previous research showed that exposure to stressful academic events is associated with higher DHEA concentration, as measured in human fingernail samples [29]. However, another pilot study compared hair DHEA levels between subjects who stated a mental burden during the previous three months and those who did not report mental burden and found no evidence of association between mental burden and DHEA concentration [30].

To date, the vast majority of studies has examined the relationship between chronic stress and the prevalence of CVD or CVD risk factors, either by using subjective stress indicators or measuring only the cortisol concentration in hair samples. The current study aimed to investigate the association between long-term secretion of stress-related steroid hormones, including cortisol, cortisone and DHEA, and 10-year fatal and non-fatal CVD risk estimated by SCORE2 risk prediction algorithm, as well as conventional CVD risk factors in a group of apparently healthy 50- to 64-year-old women. Additionally, we analyzed the concordance between hair steroid hormone levels and self-reported stress-related measures, including perceived stress, anxiety and sleep quality.

## 2. Materials and Methods

### 2.1. Study Participants

The open-access program G*Power (version 3.1) was used to calculate the study sample size. After selecting the research significance level α = 0.05 and the research power 80%, the preliminary size of the research sample was estimated. The chronic stress prevalence parameter was assumed to be 50%, as the exact prevalence of this indicator in the study population is unknown. As such, the cross-sectional study included 145 women (aged 50–64 years) participating in the national cardiovascular disease prevention program. Exclusion criteria were the presence of acute and chronic diseases, including cardiovascular disease, diabetes mellitus and chronic kidney disease, as well as mental diseases and the use of synthetic steroid hormones during the previous 3 months. Participants provided written informed consent before entering the study. The study protocol was approved by the Vilnius Regional Biomedical Research Ethics Committee (No. 2020/8-1254-735). The procedures used in this study adhere to the tenets of the Declaration of Helsinki.

### 2.2. Sociodemographic, Lifestyle and Health-Related Characteristics

Each enrolled individual was asked to fill out a questionnaire to gain information about their sociodemographic (i.e., age, education level, marital status), health-related characteristics (i.e., menopause, physically and psychologically traumatic events during the previous 3 months), lifestyle factors (i.e., smoking status, physical activity level) and hair washing frequency. Studies investigating the influence of hair washing frequency on hair steroid hormone levels showed inconsistent results: Kristensen et al. [31] found no evidence of association between frequency of hair washing and hair cortisol concentration, while Staufenbiel et al. [32] reported that individuals who wash their hair three or more times a week have lower hair cortisol and cortisone levels compared to persons washing their hair twice a week or less. Due to the aforementioned mixed findings, we included hair washing frequency among the factors potentially affecting hair steroid hormone concentrations.

Participants’ physical activity level was assessed in different domains, including physical activity at work and leisure time physical activity. Each physical activity domain was measured by a four-category rating scale (score 1—lowest physical activity, score 4—highest physical activity). Score 1 of physical activity at work was described as “mainly sedentary work, e.g., sitting at the table”; score 2—“necessary to walk during work without lifting of heavy items, e.g., work with the clients”; score 3—“intensive walking during work, carrying heavy items, e.g., post-man’s work”; score 4—“heavy, intense physical work, lifting of heavy items, e.g., work of a nurse”. Score 1 of physical activity at leisure time was described as “the time mainly spent for reading, watching TV or theatrical performances, etc.”; score 2—“minimum 4 times per week going for a walk or wheeling, light work in the garden, fishing, etc.”; score 3—“the time mainly spent for running, swimming, tennis or other similar sport”; score 4—“mainly heavy training, running, swimming, skiing, etc., regularly in matches”. Subjects were asked to rate their physical activity by selecting one statement from four to best describe their physical activity. The overall physical activity score was obtained by summing up the scores of both physical activity domains. Thus, a possible physical activity score could range from 2 to 8.

### 2.3. Stress, Anxiety and Sleep Quality Measures

Psychosocial stress level was evaluated using the 10-item version of the Perceived Stress Scale (PSS), designed to measure the degree to which situations in a subject’s life are appraised as stressful. Possible total PSS scores range from 0 to 40, with higher scores indicating a higher perceived stress level. PSS scores ranging from 0 to 13 are considered low stress, 14–26—moderate stress and 27–40—high stress [13]. Anxiety level was assessed by the trait scale of State-Trait Anxiety Inventory (STAI-T). The STAI-T scale consists of twenty statements that assess how a person generally feels. Overall STAI-T scores range from 20 to 80, with higher scores indicating a greater trait anxiety level [33]. The Pittsburgh Sleep Quality Index (PSQI) was used to evaluate a subject’s sleep quality and disturbances over the period of one month. The PSQI index consists of 19 questions, and the global PSQI index score is calculated by summing up the scores from seven different components, including subjective sleep quality, sleep latency, sleep duration, habitual sleep efficiency, sleep disturbances, use of sleeping medications and daytime dysfunction. The global PSQI index score ranges from 0 to 21, with higher scores indicating worse sleep quality. It is estimated that a global PSQI index score lower than 5 indicates good sleep quality, while a value equal to or higher than 5 means poor sleep quality [34].

### 2.4. Anthropometric Parameters, Biochemical Analyses

Anthropometric parameters (height, weight, waist circumference), resting arterial blood pressure (systolic and diastolic), and heart rate values were assessed by trained personnel. Additionally, blood samples were collected under fasting conditions and lipid metabolism biomarkers (i.e., total cholesterol, HDL-cholesterol, LDL-cholesterol, triacylglycerols), and glucose concentrations in blood serum were analyzed using routine methods (Architect ci8200, Abbott, Chicago, IL, USA) in the Centre of Laboratory Medicine of Vilnius University Hospital Santaros Klinikos.

### 2.5. Cardiovascular Risk Evaluation

Cardiovascular risk was evaluated using the recently updated SCORE2 prediction model, which estimates 10-year fatal and non-fatal CVD risk in individuals without previous CVD or diabetes, aged 40–69 years, in Europe. The SCORE2 risk prediction algorithm was derived utilizing individual-participant data from 45 cohorts in 13 countries (677,684 individuals; 30,121 CVD events). Distinct SCORE2 charts are used for low, moderate, high and very high CVD risk regions based on region-specific CVD incidence and mortality. Since Lithuania is assigned to the very high risk region, we used the SCORE2 chart developed for populations with a very high CVD risk. In general, the SCORE2 risk prediction algorithm is gender-specific and consists of age, systolic blood pressure and non-HDL-cholesterol measures. The possible SCORE2 values for 50- to 64-year-old women in the very high CVD risk region could range from 4% to 39%, with 4% indicating low CVD risk, 5–9%—moderate, ≥10%—high [2].

### 2.6. Hair Steroid Hormone Analysis

Hair samples were collected from the posterior vertex region of the head, as close to the scalp as possible. Samples were stored in foil at room temperature in a dark environment until the analysis. The proximal 3-cm hair segment, reflecting the most recent 3 months, was used for determination of the hair steroid hormone (cortisol, cortisone, dehydroepiandrosterone) levels. A sample preparation procedure was performed using a slightly modified method, as published in our previous paper [35]. Quantitative steroid hormone analysis, utilizing an ultra-high-performance liquid chromatography-tandem mass spectrometry (UHPLC-MS/MS) system, was carried out at the laboratory of the Department of Human and Medical Genetics, Institute of Biomedical Sciences, Faculty of Medicine, Vilnius University.

### 2.7. Statistical Analysis

Statistical analysis was performed using the R software (version 4.0.3). The Shapiro–Wilk test was used to test the normality of variables. Quantitative variables are presented as mean ± standard deviation (SD) for normally distributed, or median (interquartile range) (IQR) for non-normally distributed, variables. For categorical variables, absolute and relative frequencies were calculated. Spearman’s rank coefficient was used to quantify the strength of the correlation between hair steroid hormones and subjective stress, anxiety and sleep quality indicators, as well as cardiovascular risk factors and SCORE2 risk model values. For comparison of the median (IQR) values of the hair steroid hormone levels among the groups, based on the presence of factors potentially affecting hair steroid hormone levels, lifestyle characteristics or SCORE2 index values, a nonparametric Mann–Whitney U test or Kruskal–Wallis test was performed. The level of statistical significance was set at 0.05 for two-tailed testing.

## 3. Results

### 3.1. Study Group Characteristics

The results of the self-reported questionnaire showed that the majority of women were married (73.8%) and attained a level of higher education (84.1%). The analysis of lifestyle indicators revealed that almost 90% of study participants were non-smokers, but more than half of the women used to be physically inactive. Sociodemographic, lifestyle and health-related characteristics of the study group are presented in Table 1.

The analysis of subjective stress level showed that the majority of individuals perceive their lives as non-stressful or feel a moderate stress level (35.9% and 60.7%, respectively). The global score of PSQI indicated that nearly half of participants (42.0%) are poor sleepers. According to the manual for the State-Trait Anxiety Inventory [33], the average STAI-T score in a group of 389 healthy women aged 50–69 years was 31.79 ± 7.78. In our study group, 80.7% of women reported higher than average STAI-T score values, indicating an increased anxiety level in the majority of study participants. The descriptive statistics of the self-reported stress, anxiety and sleep quality indicators are presented in Table 2.

The descriptive statistics of anthropometric variables, glucose and lipid metabolism biomarkers, as well as hair steroid hormone levels are depicted in Table 3. The target values for anthropometric and blood pressure measures, as well as for lipid metabolism biomarkers, are reported in the 2021 ESC Guidelines on cardiovascular disease prevention in clinical practice [2]. The results showed that 39.3% of women had a normal body mass index (18.5–24.9 kg/m^2^), 27.6% were overweight (25.0–29.9 kg/m^2^) and 33.1% were obese (≥30 kg/m^2^). Similarly, the majority of subjects (69.7%) had higher than recommended (≥80 cm) waist circumference values. There was no evidence of hypertension in 67.6% of women, while 32.4% had increased systolic (≥140 mmHg) and/or diastolic blood pressure (≥90 mmHg). Furthermore, the results revealed that for the majority of participants (82.8% and 75.9%, respectively), LDL-cholesterol and non-HDL cholesterol levels were above the target values (2.6 mmol/L and 3.4 mmol/L, respectively).

### 3.2. Hair Steroid Hormones and Cardiovascular Risk Factors

In the first stage of the analysis, we compared steroid hormone levels between the subject groups, based on the presence of factors potentially affecting hair steroid hormone concentrations. The results showed that no statistically significant differences in hair cortisol, cortisone and DHEA concentrations were found between menopausal and non-menopausal women. Moreover, neither the experience of physically or psychologically traumatic events during the previous three months, nor hair washing frequency, had a significant impact on hair steroid hormone levels. No significant differences in hair cortisol, cortisone and DHEA levels were found among participants with different smoking statuses and physical activity levels (Table 4).

**Figure 1:**
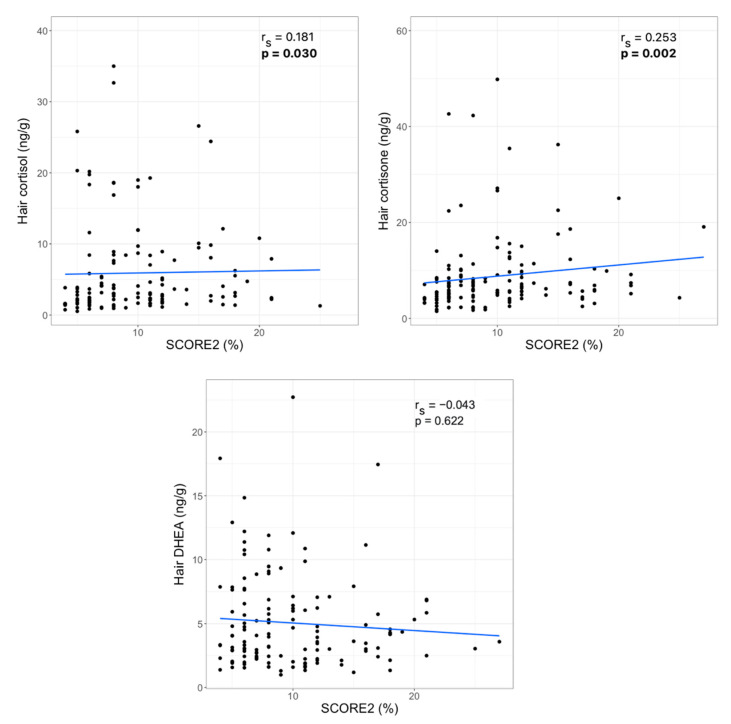
Correlations between hair steroid hormone levels and SCORE2 index (r_s_—Spearman‘s correlation coefficient, statistically significant *p*-values are bolded).

To examine the association between hair steroid hormone levels and cardiovascular disease risk, a SCORE2 index calibrated for very high-risk regions was calculated. SCORE2 estimates 10-year fatal and non-fatal CVD risk in individuals without previous CVD or diabetes aged 40–69 years in Europe. The median (IQR) SCORE2 index value in the study group was 8 (6)%. Correlation analysis revealed a statistically significant relationship between hair cortisol, as well as hair cortisone levels, and the SCORE2 index values. No significant association between hair DHEA concentration and the SCORE2 index was observed (Figure 1).

Results showed that only 4% of participants had relatively low risk (SCORE2 < 5%); moderate risk (5% ≤ SCORE2 ≤ 9%) was determined for 50% of women, and 46% of subjects had high CVD risk (SCORE2 ≥ 10%). Since the majority of women had moderate or high CVD risk, we divided the entire study sample into two CVD risk groups: the low or moderate risk group (SCORE2 < 10%) vs. the high-risk group (SCORE2 ≥ 10%). Comparison of the hair steroid hormone median (IQR) values between the low or moderate and high CVD risk groups revealed statistically significant differences in hair cortisone concentration (5.83 (3.86) ng/g vs. 7.16 (7.08) ng/g, *p* = 0.002) and no significant differences in hair cortisol (3.07 (6.48) ng/g vs. 4.07 (7.32) ng/g, *p* = 0.121) and DHEA levels (4.00 (5.20) ng/g vs. 3.93 (3.81) ng/g, *p* = 0.626).

Further analysis was performed to examine associations between the hair steroid hormone levels and individual cardiovascular risk indicators, including age, anthropometric characteristics and lipid and glucose metabolism biomarkers. Significant positive correlations were found between hair cortisol concentration and BMI, waist circumference, resting systolic and diastolic blood pressure, as well as apolipoprotein E concentration in blood serum. Similarly, hair cortisone was positively associated with BMI, waist circumference, systolic and diastolic blood pressure and glucose concentration and negatively related to HDL-cholesterol concentration in blood serum. Further, a weak but statistically significant relationship between hair DHEA level and arterial blood pressure was found (Table 5).

### 3.3. Hair Steroid Hormones, Self-Reported Stress, Anxiety and Sleep Quality

**Figure 2:**
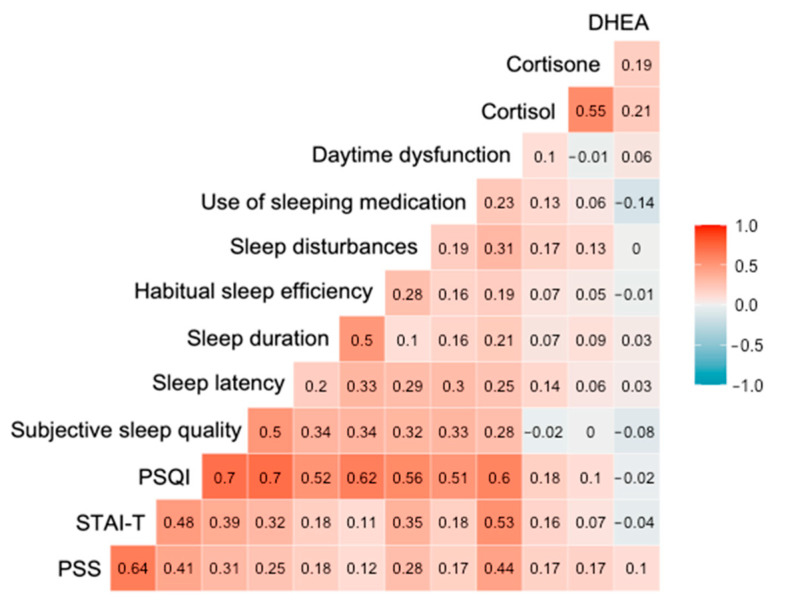
Spearman’s correlation matrix of hair steroid hormone levels, stress, anxiety and sleep quality measures (the intensity of color represents the strength of correlation).

Correlation analysis between hair steroids and self-reported stress, anxiety and sleep quality measures revealed significant associations between hair cortisol concentration and PSQI (r_s_ = 0,181, *p* = 0,030), sleep disturbances (r_s_ = 0,169, *p* = 0,043) and PSS scores (r_s_ = 0,170, *p* = 0,041). Results showed that the PSS values were significantly associated with STAI-T scores (r_s_ = 0,636, *p* = 2.20 × 10^−16^), global PSQI (r_s_ = 0.412, *p* = 2.84 × 10^−7^) and its components, including subjective sleep quality (*p* = 1.56 × 10^−4^), sleep latency (*p* = 0.002), sleep duration (*p* = 0.030), sleep disturbances (*p* = 0.001), use of sleeping medication (*p* = 0.038) and daytime dysfunction (*p* = 3.59 × 10^−8^). Similarly, STAI-T values were positively correlated with the global PSQI score (r_s_ = 0.475, *p* = 1.75 × 10^−9^) and six distinct PSQI components (i.e., subjective sleep quality (*p* = 1.70 × 10^−6^), sleep latency (*p* = 1.16 × 10^−4^), sleep duration (*p* = 0.027), sleep disturbances (*p* = 1.62 × 10^−5^), use of sleeping medication (*p* = 0.030) and daytime dysfunction (*p* = 6.23 × 10^−12^). As expected, significant intercorrelations among hair steroid hormone levels were found (i.e., cortisol and cortisone (*p* = 2.20 × 10^−16^), cortisol and DHEA (*p* = 0.013) as well as cortisone and DHEA (*p* = 0.028) (Figure 2).

## 4. Discussion

The major aim of this study was to examine the link between SCORE2 risk model values, as well as cardiometabolic risk factors, including obesity, dyslipidemia, hyperglycemia, hypertension and hair steroid hormone (i.e., cortisol, cortisone, DHEA) levels in a group of women participating in the national cardiovascular disease prevention program.

The main finding of the current study is the positive correlation between hair glucocorticoids and SCORE2 risk model values. Numerous previous studies reported an association between hair cortisol concentration and the incidence of acute coronary syndrome [36] or myocardial infarction [37,38]; elevated hair cortisol level was found to be related to the history of CVD [39], and individuals with coronary heart disease [40] or angiographically documented coronary atherosclerosis [41] had significantly higher hair cortisol levels compared with healthy controls. In contrast to these findings, a study of 3507 participants from an occupational cohort study of British civil servants showed no evidence of an association between hair cortisol concentration and the presence of coronary heart disease or the experience of stroke [42]. However, this is the first study to show that objectively measured chronic stress level is associated with SCORE2 risk model values in apparently healthy women without a diagnosis of acute and chronic diseases. Moreover, it should be noted that the Spearman’s correlation coefficient between the hair cortisone and SCORE2 was higher compared with the correlation measured between the hair cortisol and SCORE2 risk model values. Comparison of the hair glucocorticoid levels among women with ≥10% and <10% 10-year fatal and non-fatal CVD risk showed that only the hair cortisone level differed significantly between groups. These results suggest that cortisone, the inactive form of cortisol, should be considered an additional biologically relevant chronic stress marker in cardiovascular health research. In fact, chronic cortisol exposure is supposed to have an impact on the pathogenesis and progression of CVD as increased cortisol levels affect plasma lipoprotein metabolism, activate gluconeogenesis in the liver and lead to hypertension through distinct molecular mechanisms, including mineralocorticoid-induced sodium retention, plasma volume expansion and inhibition of vasodilator hormones [43]. Since cortisol is enzymatically converted to cortisone, which itself has no biological effect, it is suggested that an assessment of both hair cortisol and cortisone might better reflect the long-term HPA axis activity and chronic stress level [44].

Investigation of the anthropometric data and their relationship with hair glucocorticoid levels showed that both cortisol and cortisone concentrations were positively associated with waist circumference, but only cortisone level was significantly related to BMI. Moreover, the strength of the correlation between hair cortisone concentration and waist circumference was higher compared with the correlation between hair cortisol level and waist circumference. This is in concordance with the results of a recently published meta-analysis [45], where 146 cohorts with 34,342 individuals (aged 53.3 ± 18.4 years) in total were analyzed, and significant correlations between hair cortisol or cortisone and BMI, as well as WC, were found. The results of the aforementioned study also indicated that the strongest correlation and the largest effect size were found between hair cortisone level and waist circumference [45]. A possible explanation of hair cortisone being a better predictor of adiposity is that the level of cortisone is assumed to be more stable in the hair shaft and is not affected by the presence of a local HPA-like axis in the hair follicle [43,45].

Our results showed significant associations between all measured steroid hormones and arterial blood pressure values. Conflicting results were found in previous studies investigating the relationship between hypertension and hair cortisol or cortisone levels. A recently published paper [41] reported that the median hair cortisol concentration was significantly higher in hypertensive individuals compared with normotensive subjects. Similarly, Bautista et al. [46] showed that individuals with a higher hair cortisol concentration (above the median of the study sample) were twice as likely to be hypertensive than those with a lower hair cortisol level (below the median of the study sample). A meta-analysis based on findings from 124 (sub)samples [47] identified that only systolic blood pressure was positively associated with hair cortisol concentration, but the relationship between hair cortisol and diastolic blood pressure was overall non-significant. The results of the study conducted by Kuehl et al. [48] were in concordance with the aforementioned findings and, additionally, showed that hair cortisone concentration was not related to either systolic or diastolic blood pressure. In contrast, results of the bivariate Pearson’s correlation analysis in a group of 1258 employees of a large aerospace company showed that both their hair cortisol and cortisone levels were significantly associated with mean arterial blood pressure, however, the adjustment for covariates resulted in a non-significant relationship between hair cortisol and the mean arterial blood pressure [49]. These findings are in line with the results of the present study as Spearman’s correlation coefficient was higher in the hair cortisone–systolic (diastolic) blood pressure association compared to the hair cortisol–systolic (diastolic) blood pressure relationship. To the best of our knowledge, this is the first study to examine the association between hair DHEA level and cardiovascular risk factors. The role of DHEA in hypertension is still not fully understood, but the suggested biological effect of DHEA is based on the stimulation of endothelial NO synthase phosphorylation and results in increased endothelial-derived NO production, which plays a key role as a vasodilator. Thus, DHEA treatment is supposed to be beneficial in reducing systolic and diastolic blood pressure [50]. However, the results of the Multi-Ethnic Study of Atherosclerosis (MESA) showed that a higher DHEA concentration in blood serum was related to a higher incidence of hypertension and a greater increase in blood pressure during follow-up among postmenopausal women free of hypertension at baseline [51]. The results of the present study are in line with the aforementioned findings, as our data revealed a link between hair DHEA concentration and arterial blood pressure. In contrast, Boxer et al. [52] highlighted that six-month oral DHEA supplementation for elderly women had no significant effect on the changes in cardiovascular risk factors, including dyslipidemia, arterial blood pressure, adiposity and fasting glucose level, compared with subjects receiving placebo treatment. A more recently published meta-analysis of 18 randomized clinical trials also reported that DHEA supplementation did not significantly change systolic and diastolic blood pressure, but increased lean body mass and decreased fat mass, when compared to control groups [53].

To date, most studies have focused on the relationship between hair cortisol concentration and glycated hemoglobin level or the prevalence of type 2 diabetes: the results suggested that long-term or repeated HPA axis activation is related to the elevated glycated hemoglobin level [49,54] and higher prevalence of type 2 diabetes [32,39,42,55]. In line with the majority of previous studies [48,49,56], no significant association between hair cortisol level and fasting glucose concentration was found in the current study. There are limited data on the relationship between other stress-related steroid hormones, including cortisone or DHEA, and glucose metabolism biomarkers. Our findings suggest that there is a weak but statistically significant association between hair cortisone concentration and fasting serum glucose level. These results are in agreement with a study conducted among Chinese adults where elevated serum cortisone level was found to be associated with an increased prevalence of impaired fasting glucose and type 2 diabetes in a dose-response manner [55].

An analysis of lipid metabolism biomarkers revealed only a few significant associations: hair cortisol concentration was positively related to apolipoprotein E level, and hair cortisone level was negatively associated with HDL-cholesterol concentration in blood serum. Conflicting results were reported in previous studies where positive [56], negative [49] or no significant associations [48,57] were found between the hair cortisol level and total serum cholesterol concentration. There are a very limited number of studies that have examined the relationship between hair glucocorticoids and HDL-cholesterol level. Stalder et al. [49] performed a bivariate Pearson’s correlation analysis in a large occupational cohort and showed that both hair cortisol and cortisone were negatively associated with HDL-cholesterol level (r = −0.065, *p* < 0.05, r = −0.109, *p* < 0.001, respectively). In contrast, a more recently published population-based prospective cohort study in a group of 2984 children provided no evidence of association between hair cortisol concentration measured at six years old and HDL-cholesterol level evaluated at ages six and ten years and suggested that the relationship of biological stress with cardiometabolic risk factors may develop at later ages [58]. To the best of our knowledge, only one study investigated the association between hair cortisol concentration and serum apolipoprotein profile. Specifically, the aforementioned study examined the link between hair cortisol and ApoA1 and ApoB in a group of children and adolescents aged 4–18 years and showed that hair cortisol concentration was negatively related to ApoA1 level [59]. The present study is the first work to show a weak but statistically significant correlation between hair cortisol and serum ApoE concentration. Generally, ApoE is mainly identified in triacylglycerol-rich lipoproteins to mediate the clearance of their remnants, and it is suggested that an increased ApoE concentration is related to a higher prevalence of metabolic syndrome and premature coronary artery disease. However, the clinical significance of ApoE is still under debate as three distinct isoforms, such as ApoE2, ApoE3, ApoE4, might affect cardiovascular health differently [60]. Thus, future research should be focused on the quantitative determination of ApoE isoforms and their relationship with chronic stress biomarkers.

Although hair glucocorticoids are frequently used as a chronic physical or psychological stress biomarkers [17,30], findings on the association between stress-related steroid hormone levels and self-reported perceived stress remain largely inconsistent. Interestingly, in only a few studies, hair cortisol was positively associated with the subjective evaluated stress level. O’Brien et al. [61] reported a weak but statistically significant correlation between the total subjective stress level (Perceived stress scale+ Chaos, Hubbub, and Order scale+City Stress Index) and hair cortisol level in a diverse sample with no prescreening criteria. Another work conducted in a very specific study group (people living with HIV) presented only a nearly significant positive correlation between PSS and hair cortisol, and DHEA was negatively related to stressful life events [62]. The results of other studies showed completely different findings [24,63]. For example, Ling et al. [63] found a significant negative correlation (ρ = −0.49, *p* = 0.005) between a 10-item PSS and the hair cortisol concentration in a group of 35 women from a low-income population, while another study conducted among 164 South African women of mixed ancestry reported no association between the PSS values and hair cortisol but a significant inverse hair cortisol relationship with the Connor–Davidson resilience scale score, indicating that a higher hair cortisol level is related to impaired stress-coping ability [24]. The results of the present study showed a weak but statistically significant correlation between the PSS and hair cortisol level. Although there are large interindividual differences in the person’s ability to objectively evaluate psychosocial stress level, and this could explain the discordance between the questionnaires and endocrine stress biomarkers, the current study was conducted during the COVID-19 pandemic, which is particularly stressful for the majority of individuals. Thus, the positive association between the self-reported and biological stress levels might be partially influenced by the stressful life conditions during the pandemic.

We also observed a significant relationship between hair cortisol and the total PSQI values. To date, only a few studies have explored direct association of the HPA axis activity and sleep quality measures. For instance, Wang et al. [64] reported a significant positive correlation between hair cortisol concentration and insomnia, which was diagnosed using the Athens Insomnia scale in a small study group of female employees from a secondary or tertiary hospital. Furthermore, recently published data revealed a mediating effect of the hair cortisol concentration between shift work and sleep disorders [21]. It is suggested that the association between poor sleep quality and increased HPA axis activity is bidirectional: activation of the HPA axis might lead to inadequate sleep duration, diminished slow-wave sleep and sleep fragmentation, while insufficient sleep results in elevated glucocorticoid levels [23]. Thus, our results suggest that both poor sleep quality and higher perceived stress level are associated with an increased hair cortisol concentration, which, in turn, is related to a higher cardiovascular disease risk.

The study has several limitations that need to be addressed in future research. First, the cross-sectional study design does not provide information about causality in the relationship between stress-related steroid hormone secretions and CVD risk. Second, there is no standardized and validated method, nor reference values, for the measurement and analysis of the steroid hormone levels in human hair samples [65]. Third, our study enrolled a homogenous cohort of middle-aged women, where the majority of them were married, highly educated, already at the menopausal period, non-smoking and physically inactive. The scarce number of pre-menopausal women who smoked and experienced physically or psychologically traumatic events during the previous three months made the assessment of how these factors affect the hair steroid hormone levels or CVD risk almost impossible. Thus, the findings of the study are applicable only to a population of middle-aged women and should be confirmed in more heterogenous study group including subjects spanning a wide age range with different socioeconomic status and lifestyle habits. Finally, we conducted a pilot study including 145 subjects; therefore, weak but statistically significant associations between the chronic stress biomarkers and CVD risk should be verified in a larger cohort using more complicated statistical modelling techniques.

## 5. Conclusions

Chronic stress biomarkers were found to be associated not only with individual cardiovascular risk factors, including obesity, hypertension, dyslipidemia and hyperglycemia, but also with the SCORE2 risk model which estimates 10-year fatal and non-fatal CVD risk in individuals without previous CVD or diabetes. Although our results showed that only hair cortisol was significantly associated with subjective stress and sleep quality measures, an investigation of the relationship between other hair steroid hormones and CVD risk factors indicated that hair cortisone might be an additional chronic stress biomarker that is even more strongly related to CVD risk in a group of apparently healthy women. Together, these results indicate that stress-related hair steroid hormones, especially cortisol and cortisone, might be valuable biomarkers for CVD prediction and prevention.

## Figures and Tables

**Table 1:** Sociodemographic, lifestyle and health-related characteristics of the study participants.

Variable	Median (IQR) or *n* (%)
Age (years)	55 (7)
Education	
Lower secondary	3 (2.1%)
Upper secondary	20 (13.8%)
Higher (university or non-university)	122 (84.1%)
Marital status	
Married	107 (73.8%)
Single/divorced/widowed	38 (26.2%)
Smoking status	
Smoker	16 (11.0%)
Non-smoker	129 (89.0%)
Physical activity ^1^	
Inactive (Score 2–3)	87 (60.8%)
Active (Score 4–6)	56 (39.2%)
Menopause ^2^	
Yes	118 (81.9%)
No	26 (18.1%)
Physically traumatic events during the previous 3 months ^2^	
Yes	4 (2.8%)
No	140 (97.2%)
Psychologically traumatic events during the previous 3 months ^2^	
Yes	32 (22.2%)
No	112 (77.8%)
Hair washing frequency	
≤1/week	21 (14.5%)
2–4/week	95 (65.5%)
>5/week	29 (20.0%)

^1^ Variable has missing data (*n* = 143). ^2^ Variable has missing data (*n* = 144).

**Table 2:** Subjective stress, anxiety and sleep quality indicators of the study participants.

Variable	Mean ± SD, Median (IQR) or *n* (%)	Range
PSS	15.59 ± 5.86	0–30
PSS category		
0–13 (low stress)	52 (35.9%)	
14–26 (moderate stress)	88 (60.7%)	
27–30 (high stress)	5 (3.4%)	
PSQI ^1^	5 (5.25)	1–17
PSQI category ^1^		
<5 (good sleeper)	83 (58.0%)	
≥5 (poor sleeper)	60 (42.0%)	
STAI-T	40.30 ± 8.87	18–67
STAI-T category		
<32 (lower anxiety)	28 (19.3%)	
≥32 (higher anxiety)	117 (80.7%)	

^1^ Variable has missing data (*n* = 143).

**Table 3:** Anthropometric indicators, glucose and lipid metabolism biomarkers as well as hair steroid hormone levels of the study participants.

Variable	Mean ± SD or Median (IQR)	Range
Weight (kg)	74 (22)	46–150
Height (cm)	165.40 ± 5.72	149–182
BMI (kg/m^2^)	26.81 (8.18)	17.86–55.77
Waist circumference (cm)	85 (20)	62–136
SBP (mmHg)	127 (21)	89–180
DBP (mmHg)	80 ± 9.08	59–110
HR (bpm)	76 (15)	53–135
Glucose (mmol/L)	5.39 (0.71)	4.1–9.21
Total cholesterol (mmol/L)	5.78 (1.49)	3.75–10.69
HDL-cholesterol (mmol/L)	1.64 (0.62)	0.85–3.2
LDL-cholesterol (mmol/L)	3.52 (1.32)	1.6–8.88
Non-HDL-cholesterol (mmol/L)	4.03 (1.54)	2.07–9.47
Remnant cholesterol (mmol/L)	0.53 (0.34)	0.24–1.84
TAG (mmol/L)	1.160 (0.74)	0.52–4.01
Apo B (g/L) ^1^	0.92 (0.37)	0.093–1.85
Apo A1 (g/L) ^1^	1.56 ± 0.26	0.86–2.49
Apo A2 (g/L) ^1^	0.35 (0.065)	0.22–0.57
Apo E (mg/L) ^1^	44.70 (12.32)	23.20–79.00
Apo A1/Apo B ^1^	1.62 (0.72)	0.62–17.31
SCORE2 (%)	8.00 (6.00)	4.00–27.00
Hair cortisol (ng/g) ^2^	3.43 (6.75)	0.53–117.55
Hair cortisone (ng/g) ^2^	6.65 (4.95)	1.49–73.33
Hair DHEA (ng/g) ^3^	3.97 (4.06)	0.99–22.72

^1^ Variable has missing data (*n* = 115). ^2^ Variable has missing data (*n* = 144). ^3^ Variable has missing data (*n* = 135).

**Table 4:** Comparison of steroid hormone levels in the subject groups based on the health-related factors, hair washing frequency or lifestyle characteristics.

Variable	Hair Cortisol (ng/g) (Median (IQR))	*p*-Value ^1^	Hair Cortisone (ng/g) (Median (IQR))	*p*-Value ^1^	Hair DHEA (ng/g) (Median (IQR))	*p*-Value ^1^
Menopause						
Yes	3.15 (6.91)	0.657	6.73 (4.88)	0.342	3.60 (3.84)	0.515
No	3.62 (5.97)		5.29 (5.24)		4.63 (4.55)	
Psychologically traumatic events						
Yes	3.7 (5.28)	0.586	6.89 (4.26)	0.543	4.07 (3.71)	0.883
No	3.1 (6.92)		6.43 (5.05)		3.93 (4.16)	
Hair washing frequency						
≤1/week	2.62 (2.51)	0.134	6.76 (5.12)	0.966	5.04 (4.32)	0.473
2–4/week	3.69 (6.36)		6.61 (4.23)		3.47 (3.56)	
>5/week	4.06 (16.04)		6.07 (5.86)		4.68 (5.18)	
Smoking status						
Smoker	3.58 (6.96)	0.849	7.26 (5.05)	0.257	4.07 (4.29)	0.382
Non-smoker	3.10 (3.50)		6.24 (3.42)		3.02 (2.47)	
Physical activity						
Inactive	3.66 (9.74)	0.333	6.69 (5.73)	0.362	3.58 (3.68)	0.333
Active	3.58 (4.81)		6.50 (4.07)		4.23 (4.73)	

^1^ Mann–Whitney U test (for the comparison between 2 groups) or Kruskal–Wallis test (for the comparison between 3 groups).

**Table 5:** Correlations between hair steroid hormone levels and age, anthropometric indicators and glucose and lipid metabolism biomarkers.

Variable	Hair Cortisol (ng/g)		Hair Cortisone (ng/g)		Hair DHEA (ng/g)	
	Spearman’s *r*	*p*-Value ^1^	Spearman’s *r*	*p*-Value ^1^	Spearman’s *r*	*p*-Value ^1^
Age (years)	0.125	0.134	0.143	0.087	−0.039	0.651
BMI (kg/m^2^)	0.155	0.064	0.307	**1.85 × 10^−4^**	0.002	0.978
WC (cm)	0.170	**0.042**	0.344	**2.38 × 10^−5^**	0.012	0.886
SBP (mmHg)	0.246	**0.003**	0.271	**1.03 × 10^−3^**	0.194	**0.024**
DBP (mmHg)	0.227	**0.006**	0.276	**7.98 × 10^−4^**	0.197	**0.022**
HR (bpm)	0.0003	0.997	0.130	0.122	0.023	0.795
Glucose (mmol/L)	0.124	0.139	0.177	**0.033**	0.003	0.969
Total cholesterol (mmol/L)	−0.069	0.411	−0.127	0.129	−0.090	0.299
HDL-cholesterol (mmol/L)	−0.044	0.600	−0.249	**0.003**	−0.018	0.838
LDL-cholesterol (mmol/L)	−0.010	0.234	−0.107	0.201	−0.078	0.366
Non-HDL-cholesterol (mmol/L)	−0.079	0.344	−0.080	0.339	−0.110	0.205
Remnant cholesterol (mmol/L)	0.091	0.279	0.079	0.348	−0.089	0.306
TAG (mmol/L)	0.090	0.283	0.077	0.356	−0.082	0.343
Apo B (g/L)	0.098	0.300	0.070	0.462	−0.019	0.845
Apo A1 (g/L)	0.049	0.607	−0.158	0.093	0.030	0.762
Apo A2 (g/L)	0.054	0.571	−0.126	0.180	0.018	0.857
Apo E (mg/L)	0.191	**0.041**	0.128	0.176	0.085	0.384
Apo A1/Apo B	−0.024	0.803	−0.102	0.279	0.027	0.781

^1^ Statistically significant results (*p* < 0.05) are marked by bold font.

## Data Availability

The data supporting the reported results are archived in the National Open Access Research Data Archive (MIDAS) at www.midas.lt. (accessed on 24 October 2021).
